# A narrative review of diabetic bone disease: Characteristics, pathogenesis, and treatment

**DOI:** 10.3389/fendo.2022.1052592

**Published:** 2022-12-14

**Authors:** Bo Wu, Zhaoyu Fu, Xinyu Wang, Pengcheng Zhou, Qifan Yang, Ye Jiang, Dong Zhu

**Affiliations:** Department of Orthopaedic Trauma, Center of Orthopaedics and Traumatology, The First Hospital of Jilin University, Changchun, China

**Keywords:** diabetes mellitus, diabetic bone disease, bone, pathogenesis, treatment

## Abstract

Recently, the increasing prevalence of diabetes mellitus has made it a major chronic illness which poses a substantial threat to human health. The prevalence of osteoporosis among patients with diabetes mellitus has grown considerably. Diabetic bone disease is a secondary osteoporosis induced by diabetes mellitus. Patients with diabetic bone disease exhibit variable degrees of bone loss, low bone mineral density, bone microarchitecture degradation, and increased bone fragility with continued diabetes mellitus, increasing their risk of fracture and impairing their ability to heal after fractures. At present, there is extensive research interest in diabetic bone disease and many significant outcomes have been reported. However, there are no comprehensive review is reported. This review elaborates on diabetic bone disease in the aspects of characteristics, pathogenesis, and treatment.

## 1 Introduction

With advancing age, changes in lifestyle habits, and altered dietary structure in humans, diabetes mellitus has become the third most threatening non-communicable disease, after cardiovascular diseases and malignant tumors. Diabetes mellitus is a group of metabolic diseases characterized by chronic hyperglycemia caused by multiple etiologies, accompanied by insufficient insulin secretion and impaired action (1). According to the latest statistics of the International Diabetes Federation, the total number of patients with diabetes mellitus globally has reached 530 million in 2021, and it is estimated that this number will exceed 780 million by 2045 (2). Due to prolonged course of the disease, diabetes mellitus can cause damage to multiple systems or organs throughout the body leading to complications. Several acute and chronic complications caused by diabetes mellitus have been reported (3). In addition to the more common diabetic retinopathy, nephropathy, and heart disease, diabetes mellitus can also damage the skeletal system, causing bone loss and even osteoporosis. Osteoporosis is a systemic bone disease characterized by low bone mass and bone microarchitecture destruction, resulting in increased bone fragility and susceptibility to fractures (4). Based on dual-energy X-ray absorptiometry (DXA), osteoporosis is described as a value for BMD at the femoral neck of 2.5 standard deviation (SD) or more below the young female adult mean (T-score less than or equal to -2.5 SD) (5). Osteoporosis due to diabetes mellitus, also known as diabetic bone disease, is a chronic disease which increases bone fragility and the risk of fracture due to a reduction in bone mass and damage to the microstructure of bone tissues caused by diabetes mellitus (6, 7). Diabetic bone disease is secondary osteoporosis that predisposes patients to long-term bone pain and motor dysfunction and has a higher risk of disability and fracture than those in primary osteoporosis (8). A survey of patients with T2DM (Type 2 diabetes mellitus) showed that more than 35% patients exhibited bone loss, of which approximately 20% met the diagnostic criteria for osteoporosis (9).

In this review, the relevant research before June 2022 has been summarized with an aim to systemically evaluate diabetic bone disease. For a profound and comprehensive understanding, we have assessed diabetic bone disease from the following three aspects: characteristics, pathogenesis, and treatment. Although there are many studies on the mechanism of diabetic bone disease, the information is still limited and further research is needed. Therefore, we innovatively conducted a bioinformatic analysis to elucidate the potential mechanism of diabetic bone disease and identify treatment targets.

## 2 Characteristics of diabetic bone disease

### 2.1 Alteration of bone density

A decrease in bone mass and bone density has been reported in patients with T1DM (type 1 diabetes mellitus). Xu Y et al. reported significant abnormalities in bone trabecular structure and reduced bone density in children with T1DM (10). Studies in adult patients with T1DM showed that the decrease in bone mineral density (BMD) is predominant in the femur, while a marginal decrease is observed in the vertebral BMD (11). After adjustment for age and body mass index, only postmenopausal women with T1DM have a lower BMD than that of the normal population during the same period (12). The results of several mutually independent studies suggest that BMD may be reduced, unchanged or even increased (13–15). Increased BMD in patients with T2DM is mainly found in the femur and vertebrae (16). It is believed that the increase in BMD in patients with T2DM is mostly associated with excess energy and being overweight, and the adaptive changes in the bone to carry an increased body load can also can cause an increase in BMD (17, 18). However, there is increasing evidence that the higher fracture risk described in T2DM despite the elevated mean values of BMD and T-score is due to an impaired bone quality and this could be defined as “diabetic osteopathy” (19–21). The seemingly paradoxical conclusion is made based on the alterations of bone turnover, impaired bone microarchitecture, accumulation of AGEs, muscle weakness, anti-diabetic therapy and et.al, which could increase the fracture risk of patients with T2DM (22).

### 2.2 Alterations of bone turnover

In physiological condition, bone formation and bone resorption are in dynamic balance while bone remodeling is stable; however, in pathological conditions, the rates of bone resorption is greater than that of bone formation, resulting in a decrease in bone mass (23). For patients with T1DM and T2DM, long-term bone turnover eventually cause alterations in bone mass (24). A meta-analysis of 66 studies showed that C-terminal cross-linked telopeptide and osteocalcin were significantly lower and sclerostin was significantly higher in patients with T1DM and T2DM than in controls. TRAP were significantly lower in patients with T2DM than in controls, while there was no significant difference in T1DM compared with controls (25). Some studies have shown that patients with T1DM, T2DM have simultaneously reduced osteocalcin and type I collagen carboxy-terminal peptide β special sequence in their blood, indicating low bone turnover (26, 27). Whereas trabecular parameters did not differ between groups, cortical area and width were both considerably lower in T2DM individuals (24). Dynamic indices showed considerably lower values in T2DM in all three bone envelopes, including mineralizing surface, bone formation rate, osteoid surface, and osteoblast surface (28, 29). This can be attributed to reduced bone formation due to slowed bone matrix maturation and mineralization rate caused by the hyperglycemic diabetic environment and to reduced bone resorption due to the inhibition of osteoclast differentiation by oral hypoglycemic agents such as incretin drugs (14, 30, 31).

### 2.3 Impaired bone microarchitecture

Patients with either T1DM or T2DM have an impaired bone microarchitecture. A decrease in tibial trabecular thickness and increased trabecular separation has been observed in patients with T1DM (32). HR-pQCT was performed in 51 patients with T1DM and 64 controls, researchers discovered that patients with T1DM had significantly lower cortical thickness and lower cortical vertebral BMD. A tendency toward reduced total vertebral BMD in T1DM was also seen. When compared to controls, T1DM patients’ bone stiffness and strength both significantly decreased (33). The microstructure of the bone in patients with T2DM is abnormal, mainly in the cancellous bone, with a decrease in the number of trabeculae and morphological disorders (32, 34, 35). The number of trabeculae and trabecular thickness of the femoral head is significantly lower in patients with T2DM than those in non-diabetic patients (36, 37). Thinning of the cortical bones and increased porosity are directly related to its reduced breaking load (38). A comparative study found a 3% decrease in radial cortical bone density and a 25% increase in cortical bone porosity in patients with T2DM, compared to those in the normal population (39). Found by HR-pQCT, T2DM had smaller cross-sectional area, higher cortical porosity, and lower cortical vertebral BMD at the tibia but not the radius (40).

### 2.4 Increased risk of fracture

The increased risk of fracture in patients with diabetes mellitus is a result of decreased bone density, bone microarchitectural damage, altered mechanical properties of bone materials, and diabetes-induced falls (41, 42). In T1DM, a meta-analysis showed that the patients have a significantly higher risk of fracture and hip fracture than those in normal participants, with Relative risk (RR) values of 1.88 and 4.4, respectively; women with T1DM have a higher risk of fracture than that in men, with an RR value of 5.79 (43). A meta-analysis showed that T2DM is a risk factor for vertebral fracture with an Odds ratio (OR) value of 1.35. Patients with both T2DM and vertebral fracture have a significantly increased risk of re-fracture with a Hazard ratio (HR) value of 2.42 (44). Another meta-analysis also showed an increased overall risk of fracture in patients with T2DM compared that in normal participants, with an RR value of 1.05. Patients with T2DM are at an increased risk of hip, spine, and forearm fractures, but the risk of wrist and ankle fractures is not significantly different from that in normal participants (45). Between patients with T1DM and T2DM, an increased risk of fracture and hip fracture is observed in patients with T1DM with RR values of 1.41 and 3.43, respectively (46).

## 3 Pathogenesis of diabetic bone disease

### 3.1 Insulin and insulin-like growth factor 1 deficiency

**Figure 1:**
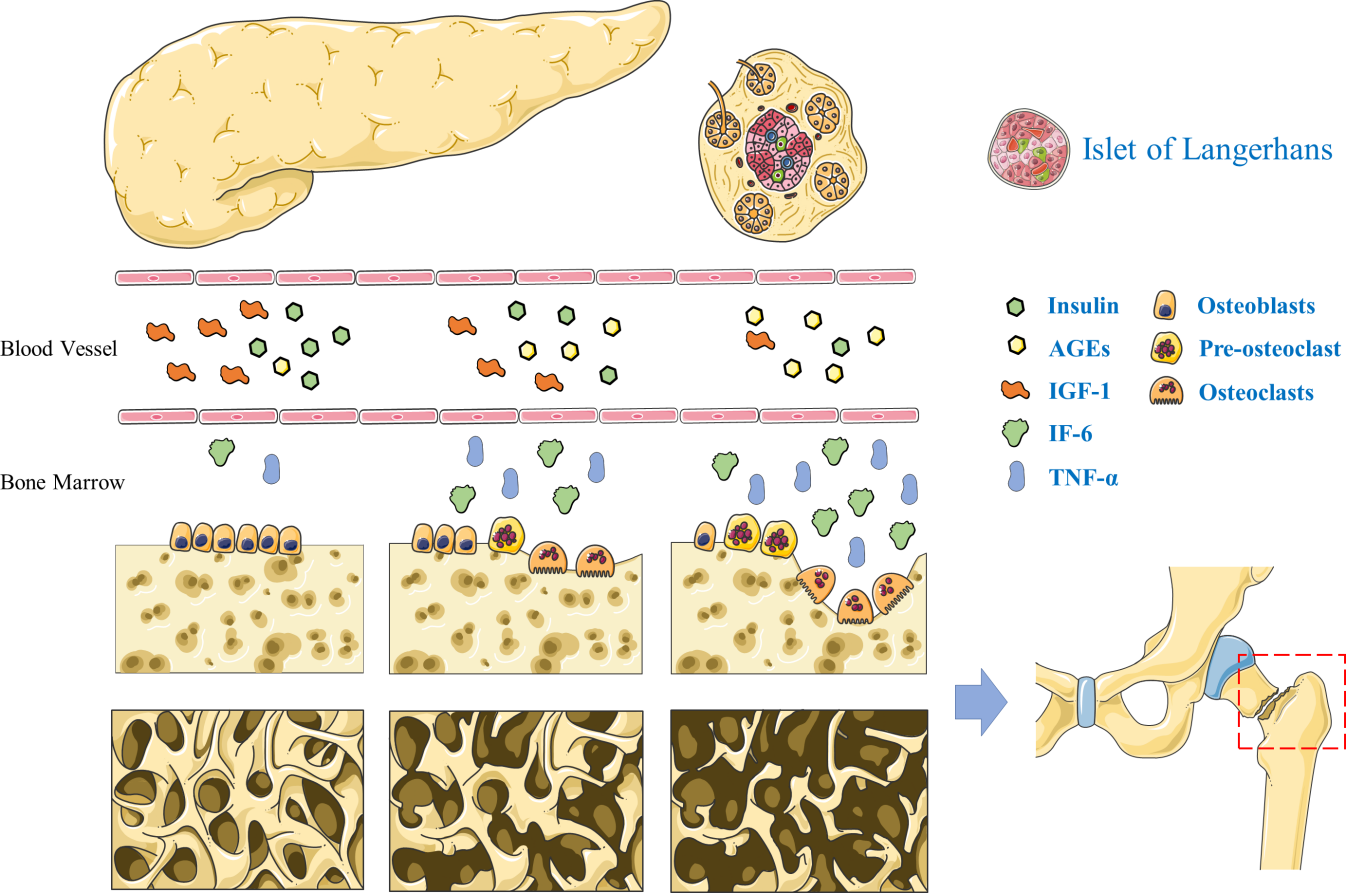
Pathogenesis of diabetic bone disease. The deficiency of insulin and insulin-like growth factor-1 leads to decreased bone formation in T2DM. Advanced glycation end products inhibited osteoblasts differentiation. Interleukin-6 and tumor necrosis factor α can promote the proliferation and differentiation of osteoclast precursor cells into mature osteoclasts and accelerate the process of bone resorption.

Insulin promotes the synthesis of various substances in biological cells. Osteoblasts rely on insulin receptors on the cell membrane surfaces to sense insulin stimulation and activate intracellular insulin signaling pathways (47, 48). Insulin can stimulate cellular DNA synthesis to induce cell proliferation and promote the synthesis of osteocalcin and collagen, which are important precursors for bone tissue formation. Insulin also promotes the expression of RUNX2, a gene related to bone metabolism, which promotes osteoblasts differentiation and bone matrix maturation (49). For patients with T1DM, the absolute deficiency in insulin secretion causes insufficient bone mineralization in adolescence due to the autoimmune destruction of pancreatic β-cells from early childhood, resulting in low bone mineralization throughout life. Patients with T2DM are characterized by systemic insulin resistance and increased feedback insulin secretion in early stage, presumably causing increased bone mineralization. In contrast, patients with advanced T2DM mellitus have decreased insulin secretion due to the decline in islet function and show a significant decrease in BMD (50, 51). Owing to its structural analogy to insulin, insulin-like growth factor 1 (IGF-1) also acts on osteoblasts to exert pro-anabolic effects, which could promote osteogenesis by accelerating collagen synthesis and bone matrix mineralization **(** **Figure 1** **)**. IGF-1 is mainly produced and secreted by hepatocytes, and patients with diabetes mellitus have significantly decreased IGF-1 levels and reduced bone formation (52). Follow-up of patients with T2DM revealed a positive correlation between serum IGF-1 levels and BMD, and reduced levels of IGF-1 is a risk factor for fracture in these patients (53).

### 3.2 Hyperglycemia and accumulation of advanced glycation end products

Hyperglycemia is the most apparent clinical manifestation in diabetes mellitus, and excess free sugars in the body adversely affect many tissues and cells. High levels of extracellular free sugars have a direct inhibitory effect on the cellular activity of, both, osteoblasts and osteocytes (54, 55). Osteoblasts exposed to high glucose levels exhibit reduced proliferation capacity, slow extracellular matrix synthesis, and subsequently slow maturation and mineralization. Thus, a hyperglycemic environment is detrimental to bone formation and the maintenance of bone mass (56). In contrast, osteoblasts show increased apoptosis and accelerated senescence in a high glucose environment. The osteocyte lacuno-canalicular system formed by osteoblasts develops diabetes-induced microstructural disruptions; the resulting abnormal bone remodeling and altered mechanical properties ultimately accelerate the development of diabetic bone disease (57–59). Moreover, a high glucose environment promotes osteoclast activation and enhances their bone resorption function (60). Advanced glycation end products (AGEs) are produced by non-enzymatic glycosylation reactions between the aldehyde groups of reducing sugars and amino acids, including carboxymethyl lysine, pentosidine, and acetonide. The abnormal accumulation of AGEs might be associated with reduced mechanical properties of the cortical bone, and the abnormal cross-linking of AGEs with collagen in the bone matrix leads to reduced collagen elasticity and ultimately to increase bone fragility (61) **(** **Figure 1** **)**. In addition, AGEs can inhibit osteoblasts differentiation and decrease alkaline phosphatase expression thereby diminishing their bone-forming effects (62, 63).

### 3.3 Pro-inflammatory cytokines and oxidative stress

Patients with diabetes mellitus are in a prolonged chronic inflammatory state, and there is prolific activation of inflammatory cytokines, including tumor necrosis factor α (TNF-α) and interleukin 6 (IL6) **(** **Figure 1** **)**. The chronic inflammatory state causes microvascular and macrovascular complications in T2DM, while adversely affecting bone remodeling (64, 65). In addition, the high glucose environment causes increased lipogenic differentiation of bone marrow mesenchymal stem cells and bone marrow fat deposition accompanied by the release of free fatty acids and large amounts of inflammatory cytokines (66). TNF-α is relatively more potent inducer promoting bone resorption. It can accelerate the bone resorption process by stimulating the proliferation and differentiation of precursor cells of osteoclasts in the bone marrow into mature osteoclasts (67). IL6 is a multifunctional cytokine secreted by a variety of human cells; it promotes osteoclast precursor cell division and proliferation, and promotes osteoclast differentiation and maturation. In addition, IL6 promotes bone resorption trough increased collagenase release and bone matrix degradation (68). Inflammatory cytokines can also cause intracellular oxidative stress. Mitochondrial aerobic respiration provide energy to cells, and during mitochondrial electron transfer, a portion of oxygen molecules are reduced to form superoxide anions, the reactive oxygen precursors (69, 70). Under physiological conditions, reactive oxygen species are involved in intracellular signaling and have a role in maintaining cellular physiological functions. However, under conditions of hyperglycemia, AGEs, and local inflammation, intracellular reactive oxygen species are abnormally increased, causing increased apoptosis of bone marrow mesenchymal stem cells, resulting in decreased bone formation (71, 72).

## 4 Treatment of diabetic bone disease

Controlling hyperglycemia is the basic treatment for diabetic bone disease, supplemented with anti-osteoporosis treatment. Specific treatment mainly includes general and pharmacological therapy. General therapy includes dietary supplements and lifestyle changes, while pharmacological therapy includes the administration of insulin, oral hypoglycemic agents, and anti-osteoporosis drugs (73). With recent advancement in research, the adverse effects of some hypoglycemic agents on bone metabolism have been gradually recognized, and the improper use of hypoglycemic agents is also considered to be associated with an increased risk of fracture in patients with diabetes mellitus. In addition, the effect of anti-osteoporosis drugs on glucose metabolism should also be considered. Thus, the combined use of hypoglycemic agents and anti-osteoporosis drugs is particularly important for the treatment of diabetic bone disease (74, 75).

### 4.1 General treatment

Patients with diabetes mellitus should increase the intake of vitamin-, mineral- and protein-rich foods in their daily diet. Deficiencies of vitamin D and calcium accelerates bone loss and is associated with insulin resistance and diabetes mellitus progression (76). Collagen synthesis requires a variety of amino acids as precursors, and insufficient protein intake is associated with reduced BMD. A meta-analysis has shown that a Mediterranean diet based on fresh fruits, vegetables, and fish can reduce the risk of fractures and microvascular complications in patients with T2DM (77). Restricting caloric intake to reduce body weight is often used to slow the progression of diabetes mellitus in obese patients; however, weight loss is assumed to be associated with reduced bone mass. Therefore, in patients with T2DM and obesity, weight control through controlled exercise is recommended (78). The bone-enhancing effect of exercise itself helps to prevent bone loss in patients with diabetes mellitus. In addition, unhealthy lifestyle habits, such as smoking and alcohol abuse should be actively avoided.

### 4.2 Insulin and hypoglycemic drugs

Hyperglycemic control is the basis of diabetic bone disease treatment. As a classic drug for diabetes mellitus treatment, insulin is related to bone metabolism. Inducing insulin signaling is essential for bone formation during growth and development. However, clinical research shows that insulin has a detrimental effect on bone loss and fracture risk in patients with diabetes mellitus. A cross-sectional survey revealed low distal radial bone density with insulin use in patients with T2DM (79). In contrast, another retrospective study showed a significant decrease in femoral neck density with 5 years of insulin use but no significant effect on lumbar spine BMD in patients with T2DM (80). In addition, one study identified insulin as an independent risk factor for hip fracture in older men with T2DM. This could be due to inappropriate insulin use, inducing an increased risk of fracture in patients due to hypoglycemic falls (81).

As the most widely used oral hypoglycemic agent, metformin slows the progression of diabetes mellitus by improving insulin resistance, reducing hepatic glucose output, and enhancing insulin sensitivity in the liver and muscle tissue. *In vitro* studies have found that the osteopathic effects of metformin are associated with its activation of the adenylate-activated protein kinase signaling pathway, and majority of the clinical studies support the osteoprotective effects of metformin in patients with T2DM (74, 82). The results of a randomized controlled study showed that increasing the daily dose of metformin from 1 g to 2 g for 3 months resulted in a significant increase in BMD in the lumbar spine and hip in older men with T2DM (83). Another study compared the effect of insulin alone and in combination with metformin to improve BMD in patients with T2DM and showed that BMD decreases significantly in patients receiving insulin alone for 18 months, while there is no significant change in BMD among patients receiving insulin combined with metformin during the same period (84). Another study proved that metformin treatment is a protective factor for all-site fracture in patients with T2DM, reducing the risk of all-site fracture by 20% compared with patients who did not receive metformin (85).

Sulfonylureas promote insulin secretion, and *in vitro* experiments have shown that sulfonylureas can exert osteogenic effects by promoting osteoblasts proliferation and differentiation (86). However, a follow-up for 104 weeks showed that glimepiride (8 mg/day) has no significant effect on systemic BMD in patients with T2DM (87). Another meta-analysis showed that patients with diabetes mellitus treated with sulfonylureas had an approximately 14% more risk of fracture compared to those who did not receive them, alongwith approximately 25% more risk of fracture compared to those treated with metformin (88).

Selective sodium-dependent glucose co-transporter 2 inhibitors (SGLT2Is) are one of the newest anti-hyperglycemic drug agents (89). A study showed that Dapagliflozin treated could rise the trabecular tissue mineral density and mean bone mineral density of tibiae using T2DM rat model (90). Canagliflozin was connected to an increase in collagen type 1, which was substantially correlated with a drop in body weight, an increase in osteocalcin, and a decrease in estrogen in females. Besides, Canagliflozin dosages of 100 and 300 mg were linked to a reduction in total hip BMD (91).

Thiazolidinediones exert their hypoglycemic effects mainly through the activation of peroxisome proliferator-activated receptor γ which regulates the insulin sensitivity of hepatocytes, skeletal muscle cells, and adipocytes. Its activation causes an increase in the adipogenic differentiation of bone marrow mesenchymal stem cells and the inhibition of osteogenic differentiation. Clinical study findings have confirmed the results of *in vitro* studies. A study of older patients with diabetes mellitus showed that the lumbar spine BMD of patients treated with thiazolidinediones decreases at a mean rate of 1.23% per year during a continuous 4-year follow-up (92). While another study has found significantly lower hip and femoral neck BMD in patients with T2DM treated with rosiglitazone compared to those who did not receive it (93). A meta-analysis showed a significantly increased risk of fracture at any body part in female patients with T2DM treated with thiazolidinediones compared to those who did not receive it, with an OR of 2.23 (94).

Incretin-based therapies are also frequently used in the treatment of diabetes mellitus. They consist of the following two main groups: Glucagon-like peptide-1 (GLP-1) agonists, such as exenatide, liraglutide, and lixisenatide, and dipeptidyl peptidase-4 (DPP-4) inhibitors, such as selegiline and alogliptin. *In vitro* studies have found that GLP-1 receptor agonists have bone-enhancing effects (95). A reticulated meta-analysis showed a significantly lower risk of fracture in patients with T2DM treated with exenatide, compared with those treated with the placebo, with an RR value of 0.17 (96). The results of another meta-analysis also confirmed that treatment with liraglutide and lixisenatide is a protective factor against fracture in patients with T2DM (97). For DPP-4 inhibitors, animal studies have shown that selegiline alleviates bone loss in diabetic rats by inhibiting bone resorption (98). A meta-analysis of clinical findings has shown a significantly lower risk of fracture in patients with T2DM treated with intractable DPP-4 inhibitors compared with those treated with the placebo, with an adjusted OR of 0.6 (99).

### 4.3 Anti-osteoporotic drugs

The commonly used anti-osteoporosis drugs function by inhibiting bone resorption or promoting bone formation. Drugs that inhibit bone resorption mainly include bisphosphonates, such as alendronate, and estrogen receptor modulators, such as raloxifene. Drugs that promote bone formation include synthetic parathyroid hormone analogs and teriparatide.

Bisphosphonates directly inhibit osteoclast activity and reduce bone resorption (100). Although patients with diabetic bone disease present a low conversion characterized by a simultaneous decrease in bone resorption and bone formation markers, anti-bone resorption drugs are still clinically effective in diabetic bone disease (101). Although alendronate has a subdued effect on increasing forearm BMD in postmenopausal osteoporosis patients with diabetes mellitus than that in postmenopausal non-diabetic bone disease patients, its effect on BMD in the hip and vertebrae is not significantly different between the two groups of patients (102). Alendronate is known to improve fasting glucose and insulin resistance in postmenopausal osteoporosis patients in preclinical diabetes mellitus (103). As a classical drug for the treatment of postmenopausal osteoporosis, estrogens with selective estrogen receptor modulators can reduce the bone resorption effect of osteoclasts by binding to estrogen receptors on their cell membranes and inhibiting osteoclast differentiation. Raloxifene showed comparable vertebral antifracture efficacy in T2DM and nondiabetic patients, while it had no effect on nonvertebral fractures in either group (104). In addition, although there was no significant effect on glycemic parameters, studies in postmenopausal female patients with diabetes mellitus confirmed that raloxifene significantly reduces serum low-density lipoprotein levels and improves lipid metabolism (105). In addition, there is a newly developed anti-osteoporosis drug targeting sclerostin such as Romosozumab, that is the first-in-class sclerostin inhibitor approved by the U.S. FDA. Romosozumab has shown excellent efficacy in the treatment of postmenopausal osteoporosis (106, 107). Additionally, new research suggests that sclerostin may play a role in the development of cancer, obesity, and diabetes mellitus (108), indicating that Romosozumab may be a promising therapeutic drug for diabetic bone diseases

Teriparatide is the first approved osteogenic drug recommended for the treatment of patients with osteoporosis and high risk of fracture. Postmenopausal osteoporosis patients with diabetes mellitus treated with teriparatide have significantly higher BMD in the femoral neck, hip, and lumbar spine than those treated with the placebo. The increase in lumbar spine BMD is 8.4% (109). A study also compared the effect of teriparatide on bone density in osteoporotic patients with and without T2DM and showed that teriparatide improved femoral neck density significantly more in patients with osteoporosis and diabetes mellitus than in those without diabetes mellitus (110). However, one study found an increase in fasting glucose levels among teriparatide-treated patients with postmenopausal osteoporosis, which may be related to an increase in intracellular free calcium concentration caused by parathyroid hormone action, resulting in a decrease in insulin-dependent glucose transporter protein and causing insulin resistance (111).

## 5 Summary

The biological basis of diabetic bone disease is closely related to a decrease in bone formation and an increase in bone resorption. A high glucose environment can significantly inhibit osteoblasts-mediated bone formation and subsequently lead to decreased bone mass. Studies about the pathogenesis of diabetic bone disease focused on insulin and IGF-1 deficiency, hyperglycemia and accumulation of advanced glycation end products, pro-inflammatory cytokines and oxidative stress, and et al. However, there are still questions, such as what’s the signaling pathways and driver genes participate in the process of bone injury under a high glucose environment? Further researches about molecular mechanism of diabetic bone disease are needed.

The treatment of diabetic bone disease depends on the control of blood glucose levels, based on which anti-osteoporosis treatment is performed, which received an un-satisfactory efficiency. Insulin injection could increase fracture risk, meanwhile, the effects of anti-osteoporosis drugs become weaker for diabetic patients. Therefore, developing new treatment strategy for patients with diabetic bone disease is promising and meaningful. In addition, the diagnosis criterion of diabetic bone disease in patients with diabetes of different ages and courses of disease is undefined and need further researches.

## Author contributions

Conceptualization, BW; investigation, ZF, XW, PZ, QY, YJ; original draft preparation, BW; review and editing, DZ. All authors contributed to the article and approved the submitted version.
